# Identification of novel source of salt tolerance in local bread wheat germplasm using morpho-physiological and biochemical attributes

**DOI:** 10.1038/s41598-021-90280-w

**Published:** 2021-05-25

**Authors:** Nadeem Hussain, Abdul Ghaffar, Zafar Ullah Zafar, Muhammad Javed, Kausar Hussain Shah, Sibgha Noreen, Hamid Manzoor, Muhammad Iqbal, Islam Frahat Zaky Hassan, Hussan Bano, Hafiza Saima Gul, Misbah Aamir, Ayesha Khalid, Younas Sohail, Muhammad Ashraf, Habib-ur-Rehman Athar

**Affiliations:** 1 Institute of Pure and Applied Biology, Bahauddin Zakariya University, Multan, 60800 Pakistan; 2 Department of Botany, University of Education, DG Khan Campus, DG Khan, Pakistan; 3 Institute of Molecular Biology and Biotechnology, Bahauddin Zakariya University, Multan, Pakistan; 4 Department of Botany, University of Okara, Okara, Pakistan; 5 National Research Center, Agricultural and Biological Research Division, Water Relations and Field Irrigation Department, Cairo, Egypt; 6 University of Agriculture, Faisalabad, Pakistan

**Keywords:** Plant physiology, Plant stress responses, Plant sciences

## Abstract

Salt tolerant wheat cultivars may be used as genetic resource for wheat breeding to ensure yield stability in future. The study was aimed to select salt tolerant cultivar(s) to identify novel source of salt tolerance in local wheat germplasm. Initially, 40 local wheat cultivars were screened at 150 mM NaCl stress at seedling stage. Selected salt-tolerant (three; S-24, LU-26S and Pasban-90) and salt-sensitive (four; MH-97, Kohistan-97, Inqilab-91 and Iqbal-2000) wheat cultivars were further evaluated using growth, yield, biochemical and physiological attributes. Growth and yield of selected cultivars were reduced under salt stress due to decline in plant water status, limited uptake of macronutrients (N, P and K), reduced K^+^/Na^+^ ratio, photosynthetic pigments and quantum yield of PSII. Wheat plants tried to acclimate salt stress by osmotic adjustment (accumulation of total soluble sugars, proline and free amino acids). Degree of salinity tolerance in cvs. S-24 and LU-26S found to be associated with maintenance of K^+^/Na^+^ ratio, osmo-protectant and photosynthetic activity and can be used as donor for salt tolerance in wheat breeding program at least in Pakistan. These cultivars can be further characterized using molecular techniques to identify QTLs/genes for salt exclusion, osmo-protectant and photosynthetic activity for molecular breeding.

## Introduction

Salinity reduces production potential of crops up to 40% and estimated crop losses are 27 billion US dollars annually^1^. To overcome economic losses, salt affected lands must bring about for sustainable agriculture by growing halophytic crops or salt tolerant glycophytic crops. Several researchers were of view to increase crop salt tolerance of our major food crops through breeding and molecular biology-based techniques^2–5^. However, success in developing salt tolerant cultivars is very poor during past five decades and it is mainly reasoned to non-availability of donor germplasm and lack of understanding about the mechanism of salt tolerance^1,6,7^.

Plant’s ability to resist toxic effects of NaCl salinity depends on genetic make-up of plants or variations in physiological processes which enable the plants to cope with salt stress, which include degree of ion exclusion, tolerance to osmotic stress and tissue tolerance^8,9^. However, detailed physiological and molecular basis of salt tolerance is poorly understood^4,10^. Moreover, mechanism of salt tolerance varies with type of species, type of cultivar of the same species, plant developmental stage which makes it more complex^4,8,11^. For example, it is well known that hexaploid wheat is more salt tolerant than tetraploid wheat, they did not differ over around 10 days of salinization^12^. Thus, screening technique and parameters used to screen and select for salt tolerance is another uphill task. This topic has been discussed in several reviews in recent years that highlight its importance and urgency^2,5,8,13–15^. These scientists were of view that identified physiological and genetic components of salt tolerance should be transferred in crop cultivars adaptive to their local environment^1,3,16,17^. Identified salt tolerant crop cultivars with physiological traits contributing in salt tolerance can be used as donor in breeding for salt tolerance. For example, Munns et al.^18^ selected a salinity-stress tolerant durum wheat line 149 having greater K^+^/Na^+^ ratio. Moreover, they used it as a potential donor line in breeding program. After introgression of this trait, they developed salinity stress tolerant durum and bread wheat lines named Nax1 and Nax2^19^. It is important to mention here that genetic variation for salinity stress tolerance in a crop species cannot be explained due to one or two traits^2,20,21^. Plant’s ability to accumulate organic osmolytes such as soluble sugars and amino acids, activation of antioxidants to scavenge reactive oxygen species (ROS), down-regulating electron transport through photosystems to lower ROS generation are some important components of salt tolerance^1,10,20,22^.

Above-mentioned reports suggest that tolerance to salt stress can be enhanced by exploiting physiological mechanism of salt tolerance. However, considerable genetic variability is pre-requisite for any specific physiological trait, based on which selection or screening can be made. Thus, the prime objective of the current study is identification of salt tolerant wheat cultivars with novel source of salt tolerance from local wheat germplasm. Moreover, it was aimed to evaluate selected wheat cultivars for salt stress tolerance using biochemical and physiological traits which can be used as potential physiological selection criteria.

## Materials and methods

### Germplasm screening experiment

The experiment was conducted at the Botanic Gardens of Bahauddin Zakariya University, Multan (30°N and 71°28E) Pakistan. Seeds of 40 locally grown wheat (*Triticum aestivum* L.) cultivars were collected from various research institutes of Pakistan including Ayyub Agricultural Research Institute (AARI), Faisalabad, Arid Zone Research Institute (AZRI), Bhakkar and Regional Agricultural Research Institute (RARI) Bahawalpur. The seeds of each cultivar were sown in rows of 10 cm apart on 1 cm thick bed of plastic beads (inert polyethene) in plastic trays (45 × 60 × 10 cm) filled with Hoagland’s nutrient solution. To salt stress group, 150 mM NaCl was also added. The 150 mM NaCl salt stress level was selected on the basis of available literature in which threshold salt concentration for different crops has been explained and considerable effect on growth has been observed. Moreover, below this concentration non-significant genotypic differences were observed. The electrical conductivity (EC) of the salt solution was measured with the help of Conductivity-meter (Jenway, 4510) and the level of solution in the trays was marked. The EC of the salt solution was measured on daily basis and maintained by adding water or salt solution up to specific point marked. After three successive hydroponic screening experiments at seedling stage, cultivars were ranked as tolerant, moderately tolerant and salt sensitive. Among salt tolerant and salt sensitive wheat cultivars, three salt tolerant and four salt sensitive cultivars were selected for further physiological assays. The selection was made based on importance of cultivars as higher yield or being largely cultivated locally.

### Adult experiment

The selected salt tolerant cultivars (S-24, LU-26S and Pasban-90) and salt sensitive cultivars (MH-97, Kohistan-97, Inqilab-91 and Iqbal-2000) were further grown for adult experimentation to identify the differences in their biochemical and morpho-physiological responses.

### Plant growth and salt treatments

Seventy plastic pots with diameter of 28 cm filled with 8 kg of well washed river sand were arranged in two groups (1) Non-saline (control) and (2) Saline (150 mM NaCl). Seeds of seven selected varieties were disinfected with 10% sodium hypochlorite solution for 10 min and rinsed with distilled water. Ten seeds of each variety were sown in pots and after seed germination, seedlings were thinned to four seedlings per pot. Thinning was done keeping in mind that seedlings were equidistantly placed and of uniform size. The experimental lay out was completely randomized design (CRD) with seven cultivars, two salt treatment and five replicates. After 21 days of germination, salt stress was applied stepwise to one group, first day 50 mM NaCl in Hoagland’s nutrient solution which was increased to 100 mM NaCl by next day and finally to 150 mM NaCl by next coming day. This practice of stepwise increasing salinity level helped to avoid sudden osmotic shock to plants. Control group received only Hoagland’s nutrient solution. Salinity stress level was maintained till the end of experiment. After four weeks of salt stress, chlorophyll content, quantum yield of photosystem II (PSII), plant water relations, accumulation of proline, total free amino acids, and total soluble proteins were measured. Two plants out of four plants were carefully harvested, roots and shoots were separated and their fresh biomass was measured. All plant parts were placed in Kraft paper bags and then dried in an electric oven at 75 °C for three days and their dry biomass was recorded. Total carbohydrates, starch, total soluble sugars and mineral nutrients were determined in oven-dried leaves. The experiment was continued with two plants per pot maintaining salinity level at 150 mM till the end of experiment and yield attributes were determined. Before the maturation of crop, the data for plant height and flag leaf area was recoded. The details of each procedure are given below.

### Measurements

#### Plant height, flag leaf area, total chlorophyll contents, and quantum yield

Plant height was taken from the soil surface to the tip of longest spike by measuring scale. Flag leaf area was calculated with the help of Muller^23^ formula i.e. maximum length × maximum breadth × 0.74. Estimation of chlorophyll content was done by a portable chlorophyll meter (Minolta, Chlorophyll meter, SPAD-502, Japan). Average of three readings from each flag leaf was taken. Middle part of the 3rd mature leaf of each plant was used to measure quantum yield of PSII by using hand held FluorPen FP-100 (Photon System International, Czech. Republic). Weak measuring light was used to measure Fo and then saturated pulse of 3000 mmol m^−2^ s^−1^ was applied to measure Fm. Using formula (Fm – Fo)/ Fm, quantum yield of PSII was calculated.

#### Plant water relations

A fully expanded leaf was used to measure leaf water potential (Ψw). The water potential in leaves were measured with the help of Scholander-type pressure chamber. Measurements for water potential were taken early in the morning to avoid variation in plant water status due to differences in water loss through transpiration. The same leaf was kept in a freezer at -20 °C for one week and then thawed by a glass rod and extracted cell sap was used for measuring osmotic potential (Ψs) by an osmometer (Vapro, 5520, USA). Turgor potential (Ψp) was calculated by the formula as Ψp = Ψw − Ψs. Leaf relative water contents were determined by weighing leaves as fresh weights after which leaves were dipped in water for 24 h and their turgid weight (Tw) was noted. These leaves were oven-dried at 80 °C for 48 h and their dried weight (Dw) was noted. Relative water content was measured as RWC (%) = [(Fw − Tw)/(Fw − Dw)] × 100.

#### Leaf proline

Proline accumulation in leaves was measured following method of Bates et al.^24^. Leaf sample (0.25 g) ground in 5 mL of 3% sulpho-salicylic acid. The grinding of leaf sample was carried out in pre-chilled pestle and mortar. The homogenate obtained was filtered. In a test tube, 2 mL filtrate was mixed with 2 mL of acid ninhydrin solution and 2 mL of glacial acetic acid. The mixture in the test tube was heated in boiling water bath for 1 h. The reaction in the test tube was terminated by placing in ice bath. Then, 4 mL of toluene was added to the terminated reaction mixture in the test tube and vortexed for 15–20 s and allowed to stand. Upper layer (chromophore) was taken and absorbance at 520 nm was recorded by spectrophotometer. The standard curve was prepared following the same procedure except using 2 mL of various standard proline solutions instead of filtered leaf homogenate. The concentration of proline in each leaf sample was measured using standard curve.

#### Total free amino acids and total soluble proteins

For the extraction of both total soluble proteins and total free amino acids, first, one gram leaf was ground in 4 mL of sodium phosphate buffer (50 mM, pH 7.8). The leaf homogenized material was centrifuged at 6000×*g* for 20 min. The supernatant was used for the determination of total soluble proteins and total free amino acids. For the determination of total soluble proteins, 20 µL sample was mixed with 2.5 mL of Bradford reagent and allowed to stand for 15 min. The absorbance of the reaction mixture was recorded at 595 nm by the double beam spectrophotometer^17^. Concentration of total free amino acids in the leaf extract was measured following the method of Hamilton and Van Slyke^25^. For this purpose, 0.5 mL sample solution, 0.5 mL of 10% pyridine and 0.5 mL of 2% ninhydrin were mixed in a test tube. The reaction mixture was heated for 30 min in boiling water bath. The reaction mixture was cooled and raised its volume up to 25 mL with distilled water. The absorbance of reaction mixture was noted at 570 nm from spectrophotometer.

#### Total carbohydrates, total soluble sugars and starch

Total soluble sugars were extracted from 0.2 g oven-dried powdered leaf material with 10 mL of 80% ethyl alcohol and repeatedly washed with 10 ml of alcohol to extract all sugars. The volume of the extract was made up to 50 mL with distilled water. The leftover residue in the test tube was diluted with 5 mL of distilled water and then added 6.5 mL of 52% HClO_4_ in it. All the test tubes were placed in refrigerator at 0 °C for 30 min and then centrifuged at 3000×*g*. The residue of each sample was washed thrice with HClO_4_. Then total volume of each sample was made 50 mL with distilled water and filtered. This filtrate was used to determine starch. For the determination of total carbohydrates, 10 mL of 6 N HCL was added to 0.2 g dried leaf material in the test tube and placed overnight. The mixture was centrifuged and the volume of the supernatant was made up to 100 mL. The leaf extract (0.3 mL) was reacted with 3 mL of anthrone solution and heated the reaction mixture for 10 min. The absorbance of the reaction was noted at 625 nm. For the estimation of total carbohydrates, starch and total soluble sugars, a standard curve of glucose solution was made.

#### Mineral elements

For the measurements of mineral nutrients in leaves, well dried and ground leaf material (0.1 g) was digested in 2 mL of digestion mixture at 250 °C on a hot plate and measured mineral nutrients as described by Allen et al.^26^. The digestion mixture was prepared by mixing 14 g of Li_2_SO_4_.2H_2_O and 0.42 g of Se in 350 mL of H_2_O_2_ in a flask kept in ice bath. Then 420 mL of conc. H_2_SO_4_ was added and stored at 2 °C. To the plant digest, 0.5 mL of HClO_4_ was added and heated till the solution become colourless. The volume of plant digest was made up to 50 mL. In the diluted digest, Na^+^ and K^+^ were estimated using flame photometer (Jenway, PFP-7). Phosphorus was measured by adding 4.5 mL of plant digest and 0.5 mL of Barton’s reagent in the test tube and allowed to stand for 20 min. Then absorption was noted at 470 nm by spectrophotometer. Nitrogen was measured by Kjeldahl’s method by taking 25 mL of plant digest, 250 mL distilled water and 4 mL of 40% NaOH in Kjeldahl flasks. The mixture was distilled until 20–25 mL distillate was collected in titration flask (almost in 30 min) containing 5 mL indicator solution. The distillate was titrated with 0.1 N H_2_SO_4_ and calculated total nitrogen.

#### Yield attributes

Number of tillers per plant, spikelets number per spike and number of grains per spike were counted. Spike length was measured by scale while grain yield per plant and 100 grain weight were measured in g with electric balance.

#### Statistical analysis

Data collected was analyzed using two-way analysis of variance (ANOVA) by statistical computer software CoStat (Version 6.303, Cohort, California, USA). The means were compared with least significant difference (LSD) test at 5% level of significance.

## Results

### Screening of germplasm

**Figure 1:**
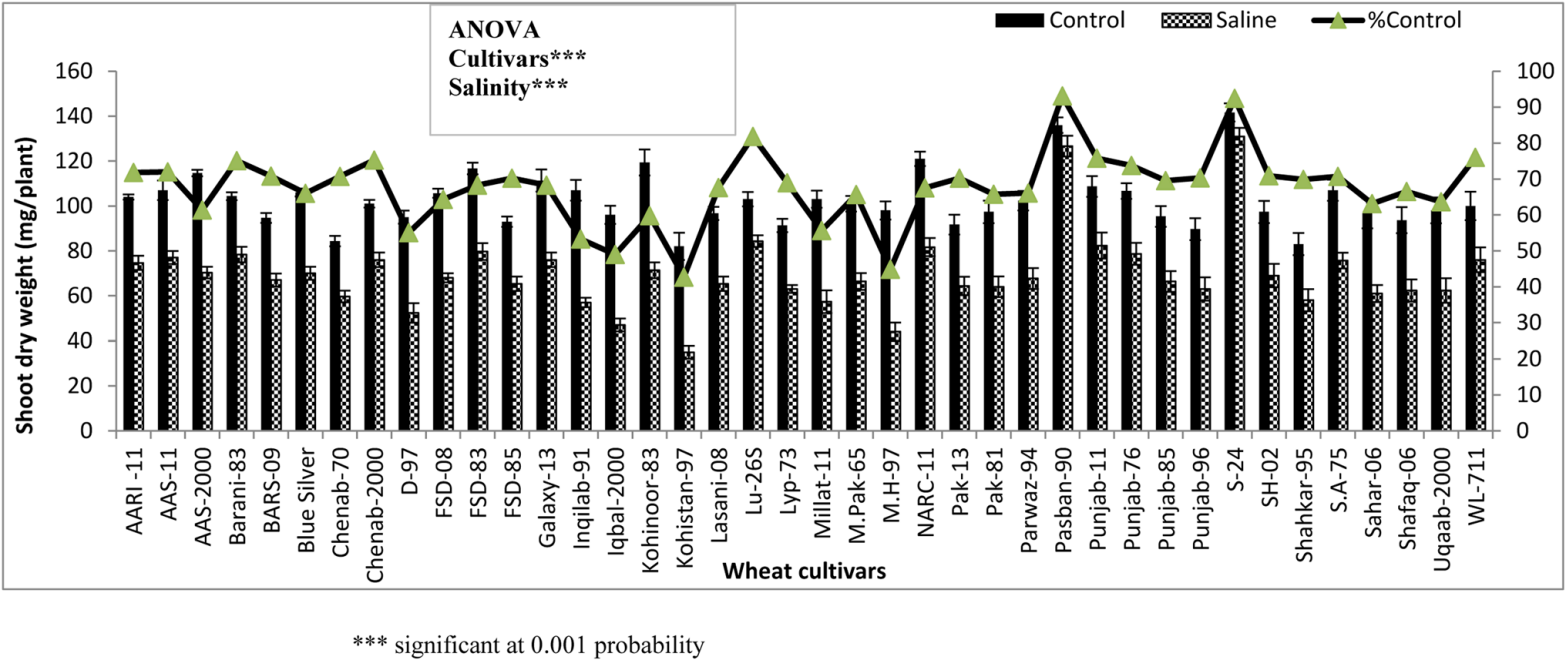
Shoot dry weights of wheat seedlings, when seeds of 40 local wheat cultivars were grown under normal or 150 mM NaCl salinity stress for three weeks. Means are presented on primary vertical axis while percent of control values are presented on secondary vertical axis.

Screening of wheat germplasm was done on the basis of growth and accumulation of mineral nutrients and given elsewhere. Only the data for shoot dry weight is given in this paper. Significant reduction (*P* ≤ 0.001) in shoot dry weight was observed in plants of all wheat varieties grown under saline hydroponic conditions as compared to control as shown in Fig. 1. Varieties Pasban-90, S-24 and LU-26S had the highest shoot dry weight under salt stress conditions, whereas varieties Kohistan-97, MH-97, Inqilab-91 and Iqbal-2000 were the lowest in this growth attribute as depicted in Fig. 1. The data for other parameters i.e. fresh biomass of shoot, fresh and dry biomass of root as well as shoot and root P, N, K^+^ and Na^+^ contents is given somewhere else.

### Adult experiment

#### Plant growth

**Figure 2:**
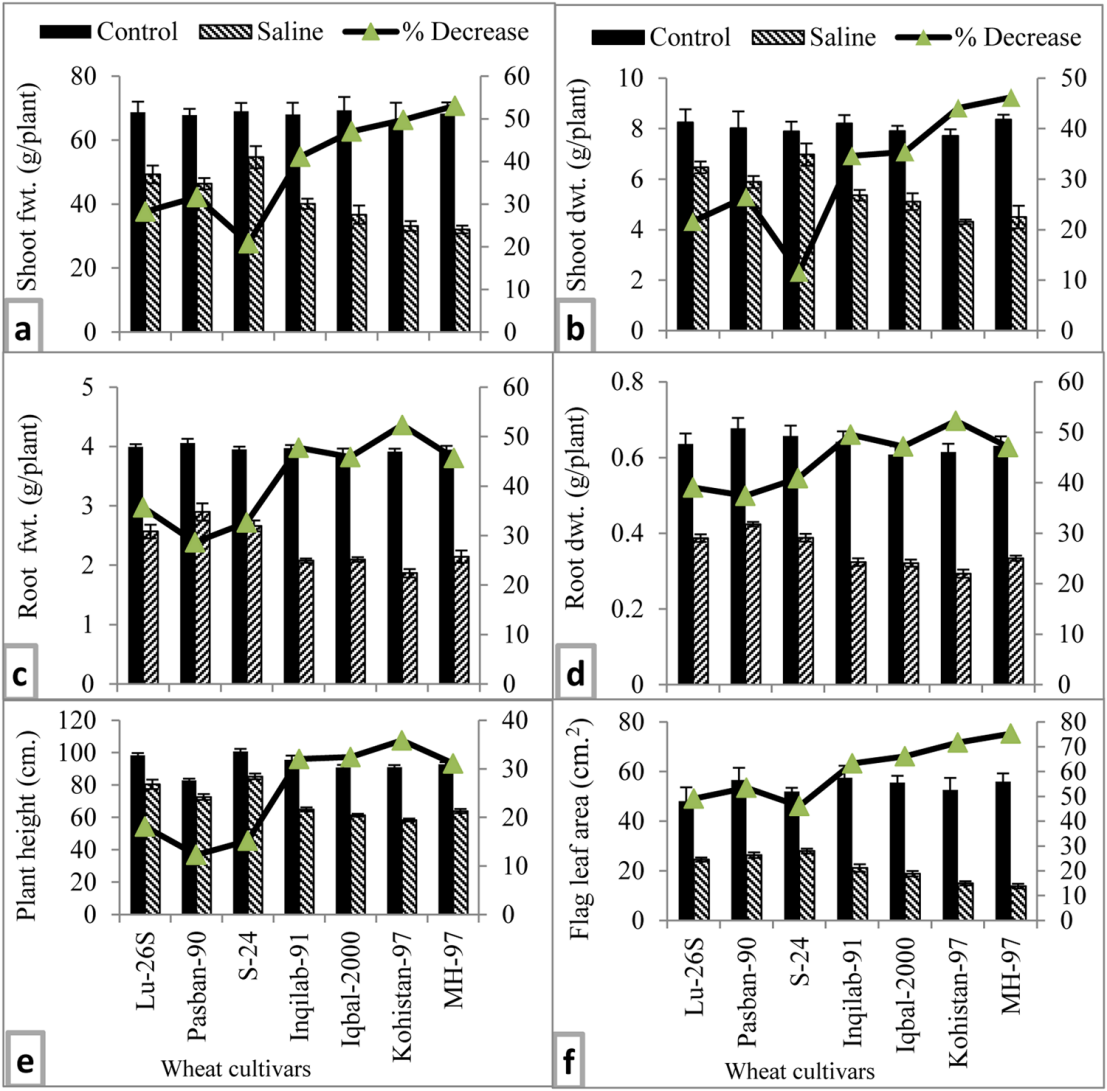
Growth attributes of selected salt tolerant and sensitive wheat cultivars at adult stage when plants of each cultivar were subjected to 0 or 150 mM NaCl salinity stress. Means are presented on primary vertical axis, while percent decrease value is presented on secondary vertical axis.

Salt stress caused a significant reduction (*P* ≤ 0.001) in shoot and root fresh and dry biomass, plant height and flag leaf area of all varieties of wheat examined in the present study (Table 1, Fig. 2). Varieties also differed significantly in all these growth attributes. The responses of these varieties to salt stress also varied significantly. For example, shoot fresh and dry biomass was reduced to ~ 50% in MH-97 and Kohistan-97 under saline conditions, while salt-induced fresh and dry biomass reduction in S-24, LU-26S and Pasban-90 was 20–30% (Fig. 2a,b). Similarly, decline in fresh and dry biomass of root was 47–52% in Kohistan-97 and Inqilab-91, while in Pasban-90, LU-26S and S-24 such reduction in root fresh and dry weight was ranged from 28 to 37% (Fig. 2c,d). Plant height of wheat varieties reduced significantly due to salt stress. Maximum reduction (37%) was observed in Kohistan-97, followed by Inqilab-91, Iqbal-2000 and MH-97, while minimum decrease in plant height due to salinity stress was found in Pasban-90, S-24 and LU-26S (Fig. 2e). Salt stress reduced flag leaf area by 71–75% in varieties MH-97 and Kohistan, whereas in S-24 and LU-26S it was reduced by 46–49% as shown in Fig. 2f.

**Table 1:** Mean squares from ANOVA of the data for growth attributes, yield parameters, photosynthetic pigment and quantum yield of PSII, water relation parameters, total soluble proteins and free amino acids, proline, total carbohydrates, starch, soluble sugars and inorganic solutes of seven local wheat cultivars when subjected to 150 mM NaCl salinity.

SOV	df	Shoot fresh weight	Shoot dry weight	Root fresh weight	Root dry weight	Plant height	Flag leaf area
Cultivars	6	208.555***	2.621***	0.450***	0.011***	489.441***	78.082*
Salinity	1	12,210.0***	112.167***	46.398***	1.419***	9704.366***	18,960.85***
Cultivars x Salinity	6	164.995***	2.526***	0.281***	0.002 ns	198.460***	128.551***
Error	56	28.930	0.386	0.019	0.001	8.549	28.491
Total	69						

SOV	df	Grain yield per plant	100 grain weight	No. of grains/spike	No. of spikelets/spike	Spike length	No. of tillers/plant
Cultivars	6	62.893***	0.885***	69.080***	17.947***	1.462**	8.128***
Salinity	1	3316.449***	26.066***	3318.914***	571.428***	457.728***	829.728***
Cultivars x Salinity	6	71.191***	0.347**	60.614***	10.995**	1.159**	8.195***
Error	56	1.834	0.113	13.2	3.185	0.346	1.557
Total	69						

SOV	df	Chlorophyll content (SPAD)	Quantum yield of PSII	Relative water content	Water potential	Osmotic potential	Turgor potential
Cultivars	6	17.859**	0.002***	88.928***	0.041***	0.102***	0.0177ns
Salinity	1	2117.5***	0.217***	2491.2***	10.414***	11.401***	0.022ns
Cultivars x Salinity	6	26.298***	0.002***	88.179***	0.059***	0.097***	0.004ns
Error	56	4.425	2.52e−4	2.004	0.007	0.003	0.011
Total	69						

SOV	df	Total soluble proteins	Total free amino acids	Proline	Total carbohydrates	Starch	Total soluble sugars
Cultivars	6	0.521***	34.209***	122.979***	98.637***	5.667***	44.413***
Salinity	1	19.575***	1544.02***	11,754.664***	22,346.822***	202.06***	3487.083***
Cultivars × Salinity	6	0.517***	60.328***	124.464***	162.587***	2.485*	42.752***
Error	56	0.031	0.857	1.560	14.828	0.876	6.326
Total	69						

SOV	df	Shoot Na^+^	Shoot K^+^	Shoot P	Shoot % N		
Cultivars	6	28.865***	27.118***	0.488***	0.338***		
Salinity	1	4460.84***	1465.013***	63.742***	32.368***		
Cultivars × Salinity	6	29.285***	7.257*	0.530***	0.364***		
Error	56	1.428	3.004	0.034	0.042		
Total	69						

*ns* non-significant, *SOV* source of variation, *df* degree of freedom.

*^,^**^,^***significant at 0.05, 0.01, and 0.001 probability.

#### Yield attributes

**Figure 3:**
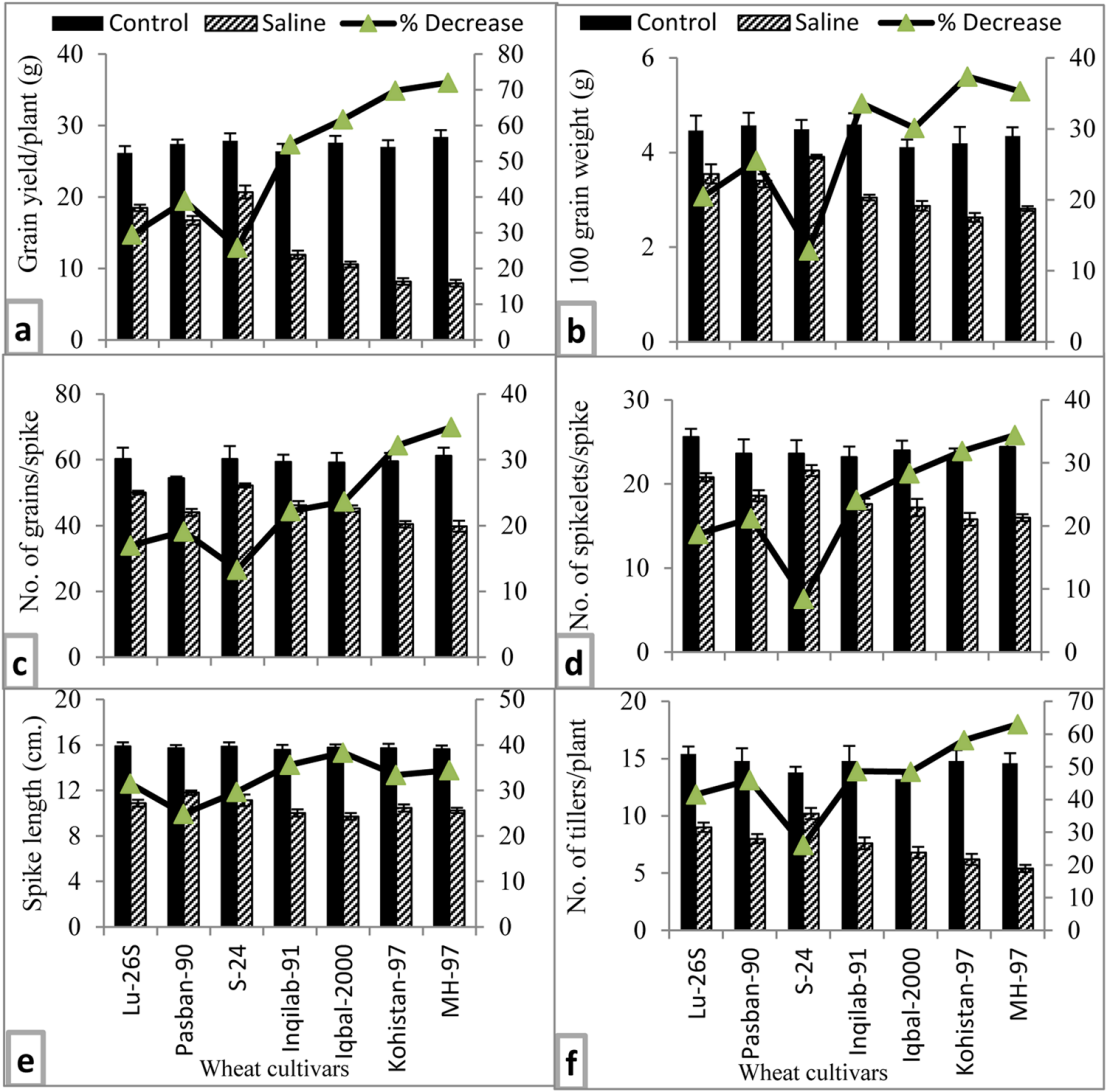
Yield parameters of selected salt tolerant and sensitive wheat cultivars at adult stage when plants of each cultivar were subjected to 0 or 150 mM NaCl salinity stress. Means are presented on primary vertical axis, while percent decrease value is presented on secondary vertical axis.

Imposition of salt stress significantly reduced yield and yield components in all wheat varieties studied in the present study (Table 1, Fig. 3). Wheat varieties differed in all these yield components under salt stress conditions. All three salt-tolerant wheat varieties had greater grain yield and 100 grain weight than those of four salt-sensitive wheat cultivars (Fig. 3a,b). Based on percent reduction, number of grains per spike were substantially decreased in salt sensitive varieties MH-97 and Kohistan-97 (Fig. 3c). Although wheat varieties did not differ in spike length under saline conditions, there was a greater percent reduction in spike length in salt sensitive wheat varieties particularly, Iqbal-2000 (Fig. 3e). Similarly, number of tillers were the maximum in salt stressed plants of salt tolerant wheat S-24 whereas the reverse was true for salt-sensitive MH-97 as shown in Fig. 3f.

#### Photosynthetic pigment and quantum yield of photosystem-II

**Figure 4:**
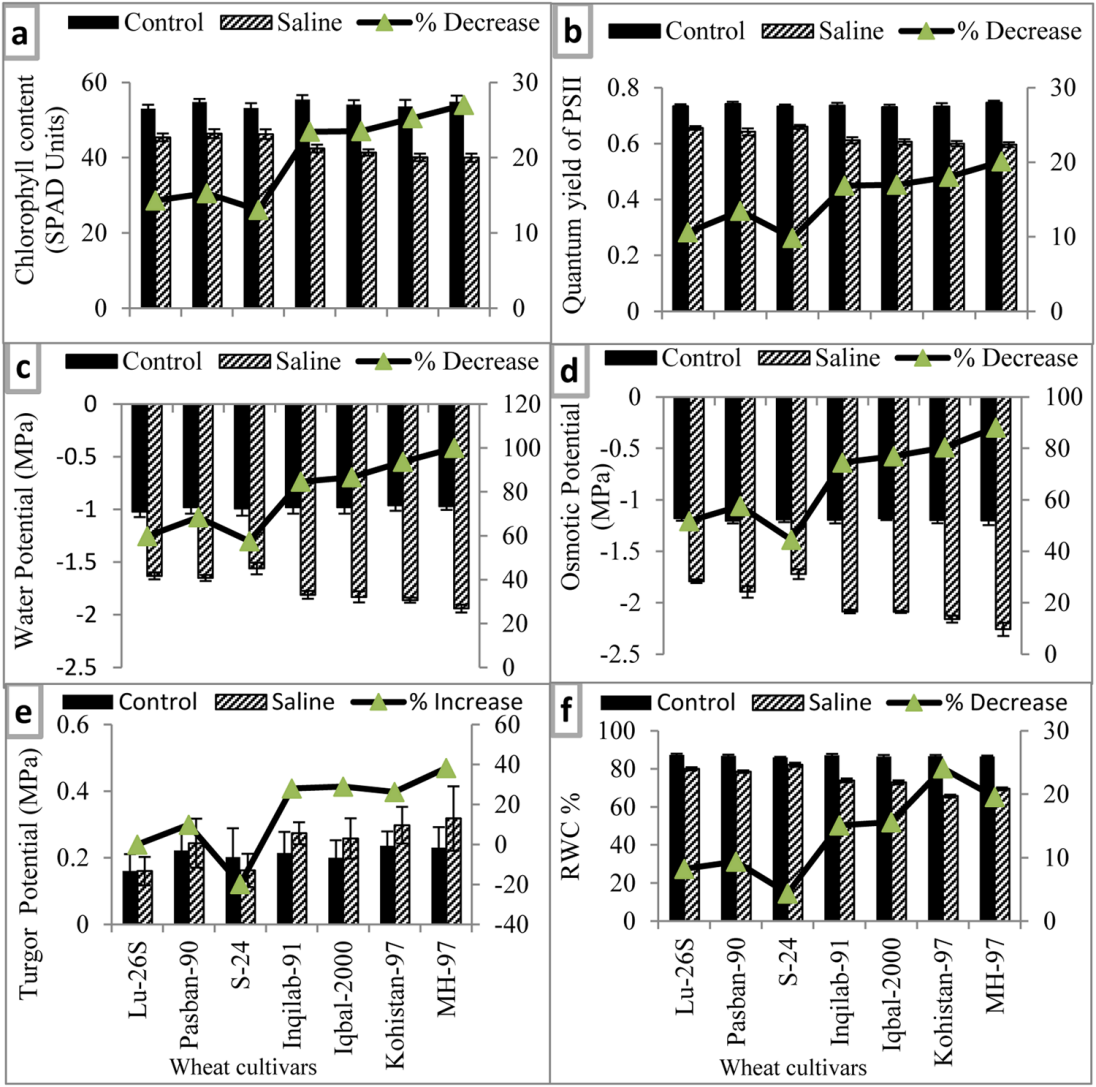
Photosynthetic attributes and water relation parameters of selected salt tolerant and sensitive wheat cultivars at adult stage when plants of each cultivar were subjected to 0 or 150 mM NaCl salinity stress. Means are presented on primary vertical axis, while percent increase/decrease value is presented on secondary vertical axis.

Total chlorophyll contents and quantum yield of PSII significantly decreased due to salt stress in all wheat varieties examined in the present study (Table 1, Fig. 4a,b). Maximum reduction in total chlorophyll measured as SPAD values was found in the leaves of salt stressed plants of MH-97 followed by Kohistan-97, whereas the reverse was true for S-24 and LU-26S. The same pattern of salt-induced reduction in quantum yield of PSII was observed in other wheat varieties (Fig. 4a,b).

#### Plant water relations

Imposition of salt stress significantly reduced the leaf water potential (Ψ_W_), osmotic potential (Ψ_S_) and relative water content (RWC), while non-significant effect on turgor potential (Ψ_P_) was observed in all wheat varieties studied (Table 1, Fig. 4c–f). All three salt-tolerant wheat varieties had greater Ψ_W_ and Ψ_S_ than those of salt-sensitive wheat varieties (Fig. 4c,d) however, tolerant and sensitive wheat varieties did not differ significantly in Ψ_P_ (Fig. 4e). Among salt-sensitive wheat varieties Ψ_W_ and Ψ_S_ were the lowest in MH-97 (Fig. 4c,d). Plant water status measured as RWC, although reduced in all wheat varieties under saline conditions, however percent reduction in RWC was maximal in salt-sensitive wheat varieties as compared to that of salt-tolerant wheat varieties (Fig. 4f).

#### Total soluble proteins, total free amino acids and proline

**Figure 5:**
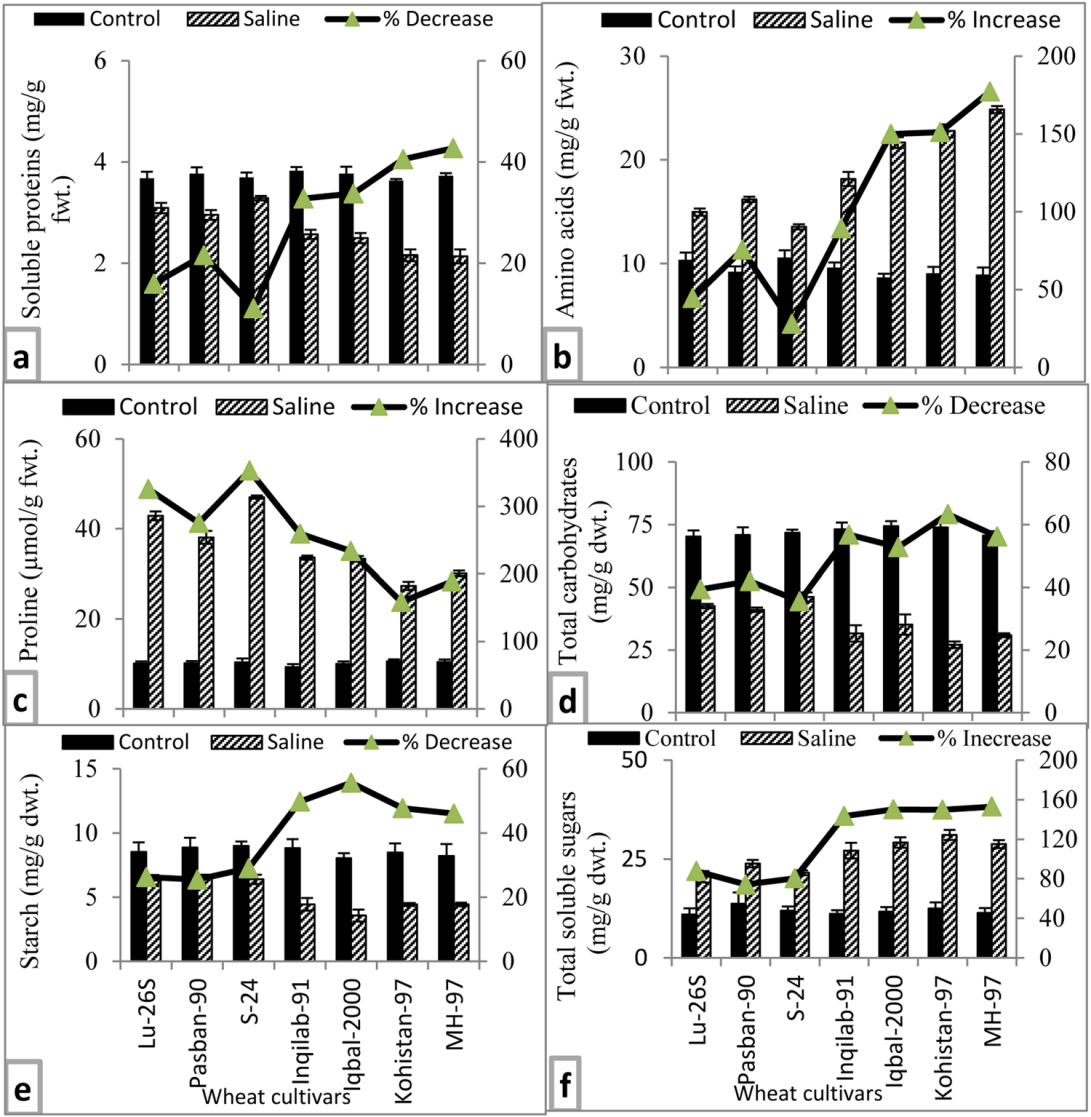
Soluble proteins, amino acids, proline, total carbohydrates, starch and soluble sugars of selected salt tolerant and sensitive wheat cultivars at adult stage when plants of each cultivar were subjected to 0 or 150 mM NaCl salinity stress. Means are presented on primary vertical axis, while percent increase/decrease value is presented on secondary vertical axis.

Growth medium salt stress caused a significant reduction in total soluble proteins, whereas total free amino acids were significantly increased in all wheat cultivars (Table 1, Fig. 5a,b). However, this reduction in total soluble proteins or increase in total free amino acids were lesser in all three salt tolerant wheat cultivars than those in four salt-sensitive wheat varieties (Fig. 5a,b). Moreover, salt sensitive MH-97 and Kohistan-97 had the lowest total soluble proteins under saline condition. In addition, maximum rise in amino acids was found in salt stressed plants of MH-97 while minimum increase was observed in S-24. Although accumulation of proline in the leaves of salt stressed plants of all wheat varieties significantly increased, maximum accumulation of proline was found in S-24 and LU-26S, whereas the reverse was true for Kohistan-97 (Fig. 5c).

#### Total carbohydrates, starch, and soluble sugars

Salt stress increased the total soluble sugars in all wheat cultivars. About 60% reduction in total carbohydrates was observed in salt sensitive varieties MH-97, Kohistan-97, while ~ 40% reduction in total carbohydrates was found in salt tolerant varieties S-24, LU-26S and Pasban-90 due to salt stress (Fig. 5d). Likewise, starch content in leaves decreased in all wheat varieties due to salt stress. However, such reduction in starch content was lower (~ 26–27%) in the salt tolerant varieties than in salt-sensitive wheat varieties (~ 45–55%) (Fig. 5e). On exposure to salinity stress of 150 mM NaCl, total soluble sugars increased substantially in all wheat varieties. However, this increase in total soluble sugars was much greater (~ 143–152%) in salt-sensitive wheat varieties than in salt tolerant wheat varieties (~ 74–87%) (Fig. 5f).

#### Inorganic solutes

**Figure 6:**
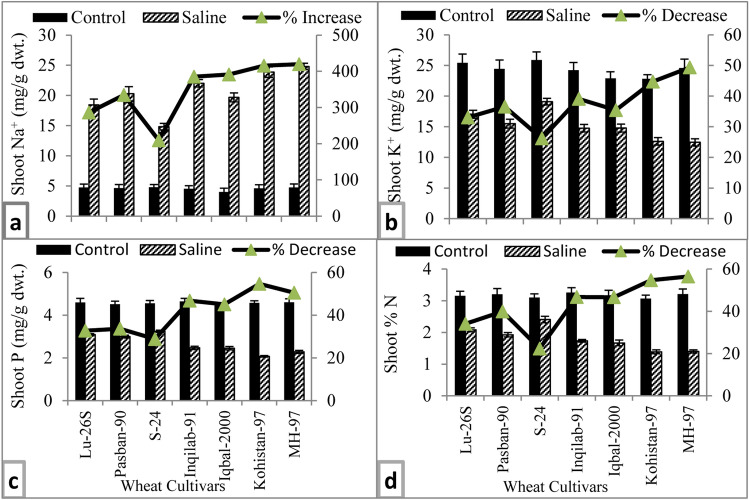
Inorganic solutes of selected salt tolerant and sensitive wheat cultivars at adult stage when plants of each cultivar were subjected to 0 or 150 mM NaCl salinity stress. Means are presented on primary vertical axis, while percent increase/decrease value is presented on secondary vertical axis.

Accumulation of Na^+^ in the leaves of all wheat varieties significantly increased due to imposition of salt stress, while accumulation of K^+^, N, and P decreased significantly (*P* ≤ 0.001) (Table 1, Fig. 6a–d). Though accumulation of Na^+^ was greater in salt-sensitive varieties than that in salt-tolerant wheat varieties, salt-sensitive variety Iqbal-2000 and salt tolerant variety Pasban-90 were equal in accumulation of Na^+^ under saline conditions (Fig. 6a). The variety S-24 accumulated the highest K^+^ in the leaves of salt stressed plants, while varieties MH-97 and Kohistan-97 were the lowest in accumulation of K^+^ in leaves (Fig. 6b). Both N and P accumulation in leaves were decreased in all wheat varieties. Salt tolerant varieties were higher in accumulation of N and P in their leaves under salt stress conditions (Fig. 6c,d).

## Discussion

Over past two decades, considerable efforts have been made to identify potential physiological selection criteria for salt tolerance in different crops, but a little success has been achieved in this regard. It is suggested in several reviews that identification of novel source of physiological component of salt tolerance and its transfer in local wheat germplasm will help to develop salt tolerant wheat cultivars. In the present study, a considerable genotypic variation for salt tolerance in a set of 40 wheat varieties at the early vegetative growth stage has been found. Three salt tolerant (S-24, LU-26S and Pasban-90) and four salt sensitive (MH-97, Kohistan-97, Inqilqb-91 and Iqbal-2000) wheat varieties were identified which is similar to earlier studies with different crop species such as in canola^21,27^, radish^28^ and wheat^18,29^. Salt tolerance can be measured as less reduction in biomass or yield in a crop cultivar under saline condition^30^. Grain yield depends on several agronomic traits, of which number of grains and size of grains are the most important^31^. In the present study, salt tolerant wheat cultivars had greater grain yield than that in salt-sensitive wheat cultivars (Fig. 3a). Moreover, genotypic difference in grain yield was due to reduction in number of grains and size of the grain. Number of grains are generally translated by tillering capacity, number of spikelets per spike and number of grains per spikelet, while size of grains are determined by translocation of photo-assimilates and plant photosynthetic activity. In the present study, significant decline in grain yield in salt sensitive wheat cultivars was mainly due to reduction in number and size of grains, and decrease in tillering capacity (Fig. 3a,c,f). These results are similar to the findings of Dugasa et al.^32^ who reported that reduction in grain yield in salt sensitive wheat cultivar was associated with decrease in tillering. Likewise, decline in grain size is another important agronomic trait contributing in reduction in wheat yield under saline condition. This can be explained as salt stress reduces availability of photo-assimilates or reduces the translocation of photo-assimilates from source to the developing grains^31,33^. These results suggested that selected salt tolerant cultivars have greater ability to produce tillers, less abortion of florets in spikelets, better ability to translocate photo-assimilates from leaves to developing grains.

Wheat growth and yield is mainly translated by photosynthetic capacity of plants and depends on amount of photosynthetic pigment, ability to efficiently convert solar energy into biochemical energy, and fixation of CO_2_ into carbohydrates. Chlorophyll content is reduced and photosynthetic process is affected in plants under salinity^34,35^. Photosystem II is damaged by salinity which is expressed as reduced quantum yield of PSII in all wheat varieties, however, S-24 and LU-26S had greater total chlorophyll content and quantum yield of PSII (Fig. 4a,b). Similar genotypic differences in quantum yield of PSII and chlorophyll content under saline conditions has already been observed in canola^35^, wheat^33^. These results suggested that such differential reduction in photosynthetic pigments and quantum yield of PSII in salt sensitive wheat varieties resulted in greater reduction in growth and yield.

Under saline conditions, the soil water potential becomes lower than water potential of plant and it can’t take up water from soil thereby reducing cell division, cell enlargement and plant growth. Plants modulate various physiological processes to maintain plant water status and growth under saline conditions such as accumulation of osmo-protectants^9,36^. In the present study, water potential reduction in wheat varieties was accompanied by lowering in osmotic potential (Fig. 4c,d) due to accumulation of organic solutes such as soluble sugars, total free amino acids, and proline (Fig. 5b,c,f) thereby maintaining a positive turgor potential (Fig. 4e). Salt-tolerant wheat varieties had greater leaf water potential, osmotic potential (less negative) and leaf RWC while salt sensitive wheat cultivars had more negative osmotic potential and lower RWC which is positively associated with greater accumulation of total free amino acids. These results suggest that salt sensitive wheat varieties were unable to effectively adjust osmotically. These results can be explained in view of the arguments of Munns^7^ that reduction in plant water status is the major cause of growth reduction under saline conditions. Moreover, greater accumulation of total free amino acids in salt-sensitive wheat varieties did not help in maintaining plant water status or in coping with adverse osmotic effects of salt stress. These results are similar to the findings of some earlier studies in which no relationship was found between accumulation of total free amino acids and degree of salt tolerance such as in sunflower^37^, safflower^38^, and four brassica species^39^.

Compatible solute accumulation for osmotic adjustment such as proline, sugar and carbohydrates under salt stress condition is an important tolerance mechanism in plants^9,35^. Greater concentration of proline plays important role in osmotic adjustment, ROS scavenging and stabilizing cellular components^7^. Although total soluble sugars, total free amino acids and proline were substantially increased in all wheat varieties due to salt stress, changes in water relations in all wheat varieties cannot be explained in view of accumulation of these organic osmotica. However, increase in total soluble sugars due to salt stress in wheat varieties was expected as salt stress cause an increase in degradation of starch or polysaccharides. Increase in accumulation of proline under salt stress is expected in view of its role as osmo-protectant and ROS scavenging activity^35^. However, relatively its greater accumulation in salt-tolerant wheat varieties could be one of the reasons of higher salt stress tolerance.

Plant salt tolerance potential depends upon the accumulation of stress responsive proteins. In the present study, salt stress caused a decline in total soluble proteins with concomitant increase in total free amino acids (Fig. 5a,b) can be explained in view of recent findings that salt stress increased the degradation of proteins by activating proteases thereby increasing concentration of free amino acids^40^. Reduction in soluble proteins under salt stress was also observed in safflower accessions^38^. However, such effect of salt stress was lesser in salt-tolerant varieties thereby relatively higher soluble proteins in salt tolerant varieties. This can be explained as biosynthesis of salt stress proteins in salt tolerant wheat varieties^10^.

In the present study, salt stress has significant detrimental effect on uptake of N, P and K^+^ contents, whereas Na^+^ accumulation increased substantially in all wheat genotypes. However, relatively lesser impact have been observed in salt tolerant varieties, particularly in S-24 and LU-26S (Fig. 6a–d). Similar results has already been observed in some of earlier studies in which it is explained that salt tolerance in mesophytes is generally associated with salt exclusion and maintenance of K^+^/Na^+^ ratio^10^. Moreover, reduced Na^+^ translocation to leaves in tolerant genotypes protects photosynthetic machinery from its toxic effects. Many studies explain that salinity tolerance can’t be predicted only from Na^+^ contents of leaves but both Na^+^ and K^+^ ions in plants determine the salt tolerance potential^7,9^.

## Conclusion

Among all wheat varieties examined in the present study, S-24 and LU-26S varieties were found to be most salt tolerant, whereas MH-97 and Kohistan-97 were found to be highly salt sensitive. Although salt tolerance in wheat varieties was associated with higher K^+^/Na^+^ ratio, and maintenance of plant water status. However, leaf turgor did not play role in degree of salt tolerance. Moreover, accumulation of total free amino acids have a role in changes in osmotic potential but did not have role in differential salt tolerance. Though accumulation of proline and total soluble sugars were greater in salt treated wheat plants, both did not have influence on leaf osmotic potential or osmotic adjustment. It is presumed, proline accumulation had role as osmo-protection. Overall, accumulation of osmo-protectants, maintaining RWC, K^+^/Na^+^ ratio, chlorophyll contents and quantum yield of PSII can be used as selection criteria in breeding for salt tolerance programs.

## Supplementary Information

Supplementary Information 1.

Supplementary Information 2.

## Data Availability

The data will be provided on request.
