# Supplementary figures and images for: Identification of novel source of salt tolerance in local bread wheat germplasm using morpho-physiological and biochemical attributes
Source: Sci Rep. 2021 May 25;11:10854. doi: 10.1038/s41598-021-90280-w (PMC8149405; doi:10.1038/s41598-021-90280-w)

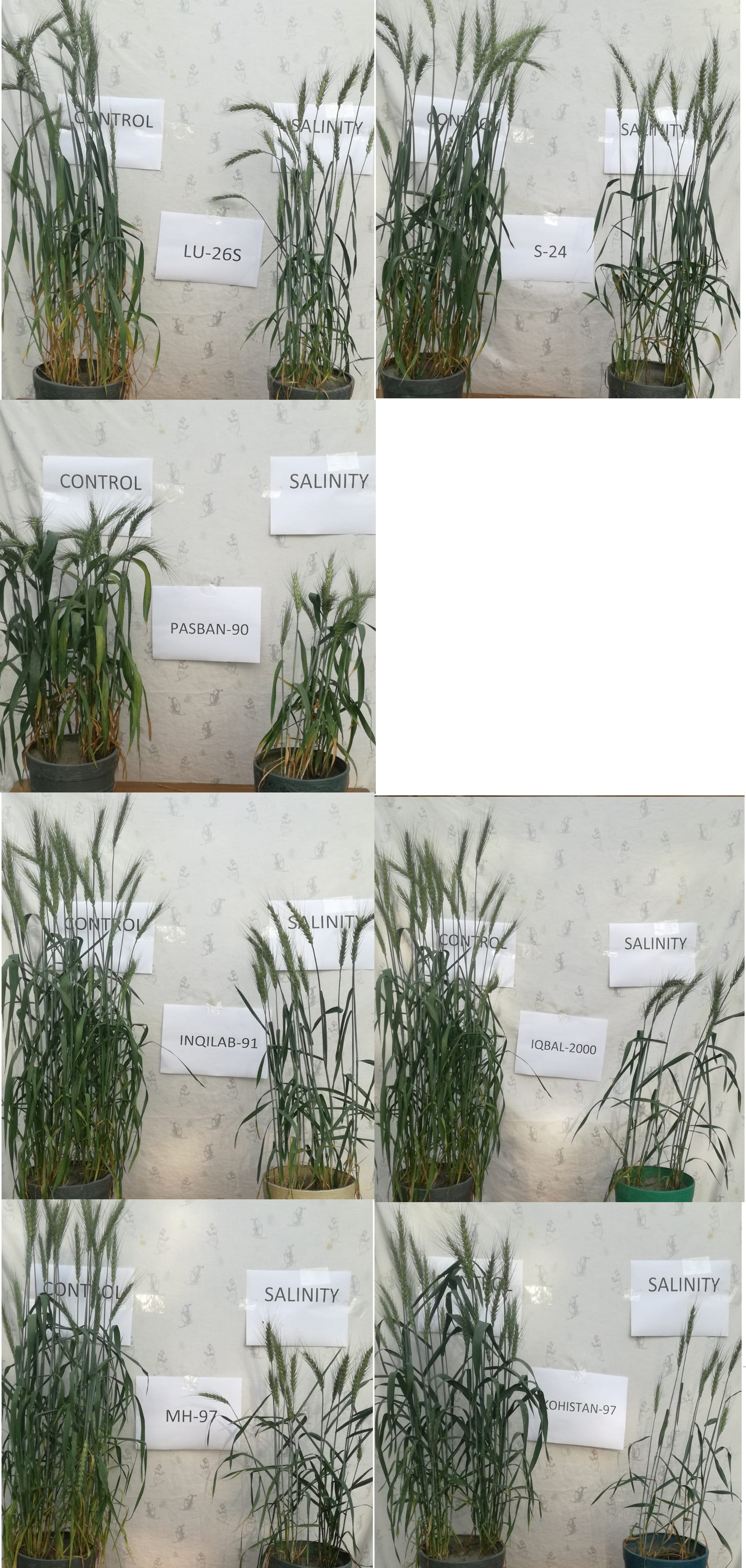

Supplement: Supplementary file 2 — Supplementary Information 2. [file 41598_2021_90280_MOESM2_ESM.jpg]
